# Optimization of tannase production by *Aspergillus glaucus* in solid-state fermentation of black tea waste

**DOI:** 10.1186/s40643-023-00686-9

**Published:** 2023-10-28

**Authors:** Moataza Mahmoud Saad, Abdelnaby Mahmoud Saad, Helmy Mohamed Hassan, Eman I. Ibrahim, Mohamed Abdelraof, Basant A. Ali

**Affiliations:** Microbial Chemistry Department, National Research Centre (NRC), 33 Bohouth St, Dokki, 12622 Giza Egypt

**Keywords:** Tannin, Tannase, Gallic acid, Residual tea, Aflatoxins test *Aspergillus glaucus*

## Abstract

Tannases are valuable industrial enzymes used in food, pharmaceutical, cosmetic, leather manufacture and in environmental biotechnology. In this study, 15 fungal isolates were obtained from Egyptian cultivated soil and marine samples. The isolated fungi were qualitatively and quantitatively screened for their abilities to produce tannase. The selected fungal isolate NRC8 giving highest tannase activity was identified by molecular technique (18S rRNA) as *Aspergillus glaucus*. Among different tannin-containing wastes tested, the black tea waste was the best substrate for tannase production by * Aspergillus glaucus* in solid-state fermentation (SSF). Optimization of the different process parameters required for maximum enzyme production was carried out to design a suitable SSF process. Maximal tannase production was achieved with moisture content of 75%, an inoculums size of 6 × 10^8^ spore/ml and sodium nitrate 0.2% (pH of 5.0) at 30 °C after 5 days of incubation. Box–Behnken experiment was designed to get a quadratic model for further optimization studies. Four-factor response-surface method with 27 runs was prepared using independent parameters including (moisture content %, initial pH, substrate concentration (g) and sodium nitrate concentration (g) for tannase model. The *F*- and *P*-values of the model were 4.30 and 0.002, respectively, which implied that the model is significant. In addition, the lack-of-fit was 1040.37 which indicates the same significance relative to the pure error. *A. glaucus* tannase was evaluated by the efficiency of conversion of tannic acid to gallic acid. Moreover, production of gallic acid from SSF process of *A. glaucus* using black tea waste was found to be 38.27 mg/ml. The best bioconversion efficiency was achieved at 40 °C with tannic acid concentration up to 200 g/L.

**Graphical Abstract:**

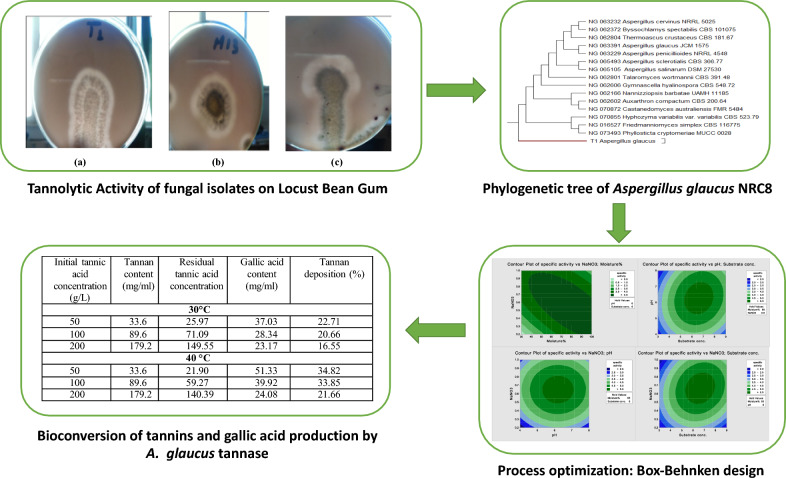

## Introduction

High rate of inflation of population, industrialization and subsequent demand for food, agricultural and aquatic resources lead to massive utilization of different environmental resources. Subsequent accumulation of byproducts, and food wastes such as husks, rotten fruits and household chores and restaurants is an additional hazard of this inflation (Govindarajan et al. 2021; Nille et al. 2021). Disposal of all these wastes into the surrounding area created a global issue of environmental pollution. Recycling of these waste streams to value-added products using simple physical, chemical or biological processes is paving a way for a clean earth.

Tea is the second most common drink after water all over the world (Hussain et al. 2018). High demand of tea results in massive tea residue left over which is discarded into the environment without prior treatment and harm human, soil, water, and environment. For instance, acidic pH of the tea waste cause soil infertility and poor farming, moreover, microbial growth on the residue leftover leads to spreading of bad odors into the surroundings. Therefore, recycling of such tea waste residues and producing value-added products are needed.

Tea waste is made up of cellulose, hemicelluloses, lignin, structural proteins and around 6.4% tannic acid (Abbiramy et al. 2015). Tannins are categorized into hydrolysable tannins, condensed tannins and complex tannins which share a combined structure between first two tannins. Catechin tannins (gallotannin or ellagitannin) are mostly found in tea leaves (Dhiman et al. 2017; Govindarajan et al. 2021). They are composed of catechin and epicatechin units linked by glycosidic linkage. Many researches are based on residual tea as substrate for microbial production of tannase (Kaur and Katyal 2018; Abd-Elmotey et al. 2022).

Tannase (Tannin acyl hydrolase; E.C.3.1.1.20) is one of the hydrolases which catalyze the breakdown of ester and depside bonds from hydrolysable tannins and gallic acid esters releasing glucose and gallic acid as hydrolysis product (Mahendran et al. 2006; Spier and Gutterres 2019; Wakil et al. 2020). Gallic acid is a phenolic acid with two intramolecular and five intermolecular hydrogen bonds. It is a significant bioactive compound due to its well-pronounced antioxidant and anti-cancer capabilities (Badhani et al. 2015). Derivatives of gallic acid have been used in various phytomedicines due to their varying pharmacological activities such as radical scavenging, cell signaling pathway interference, cancer cell apoptosis, and other activities (Belmares et al. 2004; Beniwal and Chhokar 2009; Viswanath et al. 2015; Jabbar et al. 2019).The diverse applicability of GA is associated with the merging ability of GA as pro-oxidant and antioxidant potentials (Badhani et al. 2015; Borah et al. 2023). A major problem of chemical synthesis of gallic acid is the high cost of chemicals used which additionally results low purity and low yield of GA given by acidic hydrolysis of tannic acid (Borah et al. 2023). So, GA production, by microbial tannase for hydrolysis of tannic acid, gallic acid esters, and other tannins, is considered as one of benefits of production tannase. Due to low cost of process production additionally high yield of GA (Wakil et al. 2020).

Tannases have other benefits as use in industrial field, were used in feed, food, beverages, brewing, pharmaceutical, chemical, cosmetic, leather manufacture and in environmental biotechnology (Chávez-González et al. 2012; Girdhari and Peshwe 2015; Kumar et al. 2015; Zhang et al. 2016; Cavalcanti et al. 2018). Moreover, tannase aids in the extraction of phenolic chemicals by dissolving plant matrix and inducing structural changes within bioactive phenolic molecules, such as the conversion of glucosides to aglycones (Hur et al. 2014). It can be biosynthesized from different biological sources including plants, animals, and microbes although the microbial tannase is the most common one. Microorganisms are easy to cultivate and the microbial downstream bioprocess is easier for the industrial sector (Jana et al. 2014). Microbial tannases have been produced by several microorganisms. *Aspergillus, Rhizopus, Trichoderma, Fusarium, Penicillium, Candida,* and *Saccharomyces* are the common microbial sources for tannase (Malgireddy and Nimma 2015; Cruz et al. 2017; Prigione et al. 2018; Ahmed and Abou-Taleb 2019).

The bioprocess of tannase production can be performed as liquid surface (LSF), submerged (SmF), and SSF. SmF is commonly used with bacteria, fungi and yeasts (Kumar et al. 2015; Farawahida et al. 2022).

SSF is a fermentation process in which the microbes grow in a solid medium without a free flow of aqueous medium. It is largely used for utilization of the agro-industrial wastes in the bioprocess development for the production of valuable products, such as enzymes and antibiotics, using mathematical modeling. SSF is preferred over SmF and LSF because the substrate itself acts as a carbon source in the absence or presence of free water; also due to their simple medium preparations and cultivations and low medium and moisture levels per weight of substrate (Bhargav et al. 2008; Kumar et al. 2015). Therefore, SSF offers a good chance for utilization of tea waste residues for production of high-added-value byproducts (Mukherjee and Banerjee 2006; Purohit et al. 2006; Wu et al. 2018; Ahmed and Abou-Taleb 2019; Abd-Elmotey et al. 2022).

The aim of this study was the utilization of tannin-containing wastes for large-scale production of valuable tannase and gallic acid by isolated fungi. Therefore, screening of potential fungal isolates was carried out to select the potent one able to produce tannase enzyme using different wastes as substrates. In addition, statistical design was applied for the optimization of different process parameters through preliminary one-factor-at-a-time approach to obtain the critical components of the medium. Then Box–Behnken design was applied to define and optimize the significant variables affecting the enzyme production. Moreover, production of gallic acid in SSF by *A. glaucus* using black tea waste was also evaluated.

## Material and methods

### Chemicals

Gallic acid methyl ester (methyl gallate) was purchased from Tokyo Chemical Industry, U.S. Gallic acid was purchased from Oxford laboratory, India, tannic acid was derived from AVI-CHEM., India. Rhodanine was purchased from Sigma Chemical Co., US and Potato Dextrose Agar (PDA, Condalab, Spain).

Tannin-rich wastes (maize bran, rice bran, wheat bran, olive leaves, coffee powder, residual tea, green tea, banana peels, tamarind, pomegranate peels, potato peels) were obtained from local Egyptian market. All other chemicals were of the highest analytical grade from NRC laboratory.

### Isolation of fungi

Fungal isolates were isolated locally from Egyptian cultivated soil samples (El-Sharkia), marine samples (Alexandria and Hurghada), and from residual raw materials as (residual tea and rotten pomegranate peels). Briefly, one gram of each soil sample, rotten pomegranate peels, and of residual tea powder, were left in open air for four days then added to 10 ml of sterilized distilled water then shaken on a rotary shaker for one hour at 200 rpm and 28 °C followed by serial dilution procedure to give up to 10–6 dilutions. One ml of diluted sample was spread on PDA agar plates supplemented by Strepto Penicillin (10 µg/L), and Rose Bengal (67 mg/l). For marine samples one ml from each sample was surface-plated on PDA agar plates supplemented by Strepto Penicillin (10 µg/L), and Rose Bengal (67 mg/L) (Routien 2018). After three days of incubation at 28 ± 2 °C, grown fungal colonies were picked and maintained on PDA agar slants and stored at 4 °C.

### Screening of fungal isolates for tannase production

#### Qualitative screening

Primary screening of fungal isolates for tannase production was carried out using modified Czapek-Dox agar plates containing (g/L): tannic acid, 10; NaNO_3_ 2; KCl 0.5; MgSO_4_·7H2O 0.5; KH_2_PO_4_ 1.0; FeSO_4_·7H_2_O 0.01 and Agar–agar 20 (pH 5.0) (Gustavo et al. 2006). Tannic acid was filter-sterilized using cellulose nitrate membrane of 25 mm diameter and 0.45 µm pore size; Whatman Ltd. After medium sterilization at 121 °C for 20 min, tannic acid was added at a final concentration of 1% (w/v). The plates were inoculated with fungal isolates and incubated at 28 ± 2 °C. The clear zone diameters around the fungal colonies, indicating a positive bioconversion of tannic acid to gallic acid and glucose, were measured after four days of incubation. Fungal isolates that showed clear zone were selected for a quantitative screening.

#### Quantitative screening

Quantitative screening was carried out in 250-ml Erlenmeyer flasks containing 50-ml aliquots of the modified liquid Czapek-Dox medium supplemented with 1% tannic acid. The flasks were inoculated with the selected fungal isolates (6 × 10^8^ spore/ml) and incubated at 28 °C for five days. After incubation, crude tannase (filtrate) was separated by filtration using Whatman filter paper No. 1. Tannase activity and protein concentration were estimated in the crude enzyme extract.

### Tannase production via SSF

The initial culture medium for SSF comprised 5 gm of substrate supplemented with 5 mL of liquid production medium (Czapek-Dox medium) without carbon source at the initial pH (5.0). The medium was autoclaved at 121 °C for 20 min, cooled and inoculated with 1 ml (6 × 10^8^ spore/ml) of spore suspension. Cultures were incubated at 30 °C for 5 days.

### Enzyme extraction

Crude enzyme extract containing extracellular tannase was extracted from the SSF broth. First, 50 ml of citrate buffer (0.05 mol/L, pH 5.0) was added to the flask. The flask was then maintained on a rotary shaker at 25 °C and 180 rpm for 1 h. The culture mixture was then squeezed by a cloth to separate the crude extract. The obtained supernatant was used as crude enzyme source for tannase assay (De la Cerda Gómez et al. 2011; Wu et al. 2018).

### Tannase assay

Tannase was assayed by the method based on chromogen formation between gallic acid (released by the action of tannase on methyl gallate) and rhodanine (Sharma et al. 2000). The substrate solution (0.01 M methyl gallate prepared in 0.05 M citrate buffer, pH 5.0), enzyme sample and buffer (0.05 M citrate buffer, pH 5.0) were preincubated at 30 °C for 5–10 min before the enzyme reaction start. The reaction mixture in the blank, test, and control tubes contained 0.25 ml of substrate solution, 0.25 ml of the buffer and 0.25 ml of the enzyme sample were added to the blank and test, respectively.

Both 0.3 ml of 0.667% (w/v) methanolic rhodanine and 0.2 ml of 0.5 N potassium hydroxide were added successively to each tube and incubated at 30 °C for 5 min. This was followed by the addition of enzyme sample (0.25 ml) to the reaction mixture in the control tube only. Finally, all tubes were diluted with 4.0 ml distilled water and incubated at 30 °C for 10 min. The absorbance was recorded at 520 nm using a spectrophotometer (Agilent-Cary 100, Germany). One unit of the enzyme was defined as micromole of gallic acid formed per minute.

Protein content of the samples was determined by Bradford’s reagent (Bradford 1976) using bovine serum albumin as a standard. Specific activity of tannase was expressed by the mean of enzyme activity (U) per milligram of protein (mg) of each sample.

### Detection of aflatoxins in the culture filtrate of the selected fungal isolate NRC8

Fungal isolate NRC8 showing highest level of tannase activity was obeyed to aflatoxins test. For detection, culture filtrate was treated with chloroform followed by a precipitation process with n-hexane until precipitate dissolution was performed into chloroform (Murakami and Suzuki 1970). High-performance liquid chromatography (HPLC Model 1525, USA) was then used for detection of aflatoxins using aflatoxins standards (B_1_, B_2_, G_1_ and G_2_). The HPLC system consisted of: waters Binary Pump Model 1525 (a water 1500 Rheodyne manual injector, a water 2475 multi-wavelength Fluorescence Detector), data workstation with software Breeze 2.A phenomenex C18 (250 × 4.6 mmi.d.), 5 µm from Waters corporation (USA). An isocratic system with water: methanol: acetonitrile 6:3:1 was applied. The separation was performed at ambient temperature at a flow rate of 1.0 ml/min. The injection volume (1.0 ml) was operated at wavelength of 360 nm for excision and 440 nm for emission (Deabes et al. 2012).

### Molecular identification

The most potent fungal isolate in tannase production in this study was isolated from tea waste. The selected isolate was identified genetically using 18S rRNA-based molecular technique. DNA extraction was done using protocol of Gene Jet Plant Genomic DNA Purification Kit (Thermo-Scientific, K0791, made in Germany). Primers used for PCR and DNA sequencing are ITS1 (5′-TCCGTAGGTGAACCTGCGG-3′) and ITS4 (5′-TCCTCCGCT TATTGA TATGC-3′). The PCR conditions were run as follows: initial denaturation at 96 °C for 3 min followed by 25 cycles of denaturation at 96 °C for 30 s, annealing at 55 °C for 30 s and an extension step at 72 °C for 1 min. The purified PCR product was sequenced, and these sequences were subjected to BLAST similarity analysis available from NCBI Gen Bank database. A phylogenetic tree was constructed using the Tamura-neighbor joining method by MEGA X software (Molecular Evolutionary Genetics Analysis, Bioinformatics, Tokyo Metropolitan University, Hachioji, Tokyo, Japan).

### Optimization of tannase production by *Aspergillus glaucus*

Different tannin-rich wastes (maize, rice bran, wheat bran, olive leaves, coffee husk, residual tea, green tea, banana peels, tamarinds seed powder, pomegranate peels, potato peels) were used as substrates for the production of tannase. Five gm of each substrate was taken into 250-ml Erlenmeyer flasks and supplemented with 5 ml of the production medium (pH 5.0). After sterilization, media was inoculated by one ml of spore suspension and incubated at 30 °C for five days. After incubation, tannase activity and protein content were estimated.

Other physical parameters were also investigated for the production medium (SSF), such as moisture level (25, 50, 75 and 100%); pH (4.0, 5.0, 6.0, 7.0, and 8.0); temperature (20, 25, 30, 35 and 40 °C); incubation time (3, 6 and 9 days); residual tea concentration (1,3,5,7 and 9 g); inoculum size (0.5, 1, 1.5, 2, 2.5 and 3 ml of spore suspension (6 × 10^8^ spore/ml) and nitrogen source (yeast extract, peptone, beef extract, soy bean, flax bean, NH_4_Cl, NH_4_SO_4_, (NH_4_)_3_PO_4_ and NaNO_3_).

### Box–Behnken experiment

Based on the effective factors that were clearly promote the tannase productivity through the One-Factor-At-A-Time (OFAT), Box–Behnken experiment were designed to get a quadratic model for further optimization studies. Four-factor response-surface method with 27 runs was prepared using independent parameters including, (X1) moisture ratio %, (X2) initial pH, (X3) substrate concentration g, (X4) sodium nitrate concentration % for tannase model (Table 1). A quadratic model was applied via two different ranges (low, and high) for each variable and examined the quadratic effects and central points to determine the variability with tannase production as the responses (Y). Generation of Box–Behnken design and analysis of variance (ANOVA) was carried out using Minitab 17-software (version 17.0.0).In addition, the possibility of this procedure was verified by the predicted responses resulting from RSM which were compared with the actual responses. An analysis of variance (ANOVA) using the *F* test (a value of *P* < 0.05 was considered significant), defined as the ratio between the pure error square and the lack-of-fit square was used to evaluate the significance level of the lack-of-fit to evaluate the significance and accuracy of this model. Analysis of coefficient *R*^2^ was also conducted in order to determine the significant differences between variables and evaluated the efficacy of the designed model and it must be close to 1.

**Table 1:** Box–Behnken experimental design

Factor	Name	Minimum	Maximum
*X1*	Moisture ratio %	30	100
*X2*	Initial pH	4.0	8.0
*X3*	Substrate concentration (g/L)	3.0	9.0
*X4*	Sodium nitrate concentration %	0.2	1.0

### Estimation of gallic acid in cultured broth

The gallic acid concentration in the cultured broth was estimated using the method of (Bajpai and Patil 1996). The culture supernatant of one mL was dissolved in 9 mL of acetate buffer at pH 5.0 and absorbance was measured at 254.6 nm and 293.8 nm using UV spectrophotometer. The concentration was deduced using the equation below:

$${\text{Gallic}}\,{\text{acid}}\,\left( {\upmu {\text{g/mL}}} \right)\, = \,{21}.{77}\left( {{\text{A254}}.{6}} \right)\,{-}\,{17}.{17}\left( {{\text{A293}}.{8}} \right).$$

### Bioconversion of tannins using *Aspergillus glaucus* tannase for gallic acid production

The* Aspergillus glaucus* tannase was incubated in the solution containing various concentrations of tannic acid (50 g/L, 100 g/L and 200 g/L), pH 5.0 at 28 °C under shaken conditions. The samples were drawn for gallic acid and residual tannic acid estimation (Wagh 2010). The total tannin content of each concentration was determined as a procedure described by Scalbert et al. (1989) and Abd-Elmotey et al. (2022). 0.5 ml of a tenfold diluted sample was mixed with 2.5 ml of tenfold diluted Folin–Ciocalteu’s phenol reagent. The reaction mixture wad incubated for 5 min before addition of 2 ml of 20% (w/v) Na_2_CO_3_. The mixture was incubated for 60 min at room temperature. The absorbance versus prepared blank was monitored at 760 nm.

The tannin degradation was determined using a tannic acid standard according to the following formula:

$${\text{Percentage}}\,{\text{of}}\,{\text{tannin}}\,{\text{degradation}}\,(\% )\, = \,{\text{Tannin}}\,{\text{before}}\,{\text{degradation}}\,{-}\,{\text{Tannin}}\,{\text{after}}\,{\text{degradation/tannin}}\,{\text{before}}\,{\text{degradation}}\,*\,{1}00.$$

### Spectrophotometric estimation of gallic acid

Spectrophotometric estimation of gallic acid was carried out by the method described by Sharma et al. (2000). 300 μl of methanolic rhodanine (0.667% in methanol) was added to the standard gallic acid or suitably diluted sample followed by addition of 200 μl of 0.5 M potassium hydroxide. After incubation at 30 °C for 5 min, 4 ml distilled water was added. The absorbance was read at 520 nm after 5–10 min.

## Results

### Isolation and screening of fungal isolates for tannase production

Fifteen fungal species were locally isolated from Egyptian cultivated soil samples (El-Sharkia), marine samples (Alexandria and Hurghada), and from residual raw materials as (residual tea and rotten pomegranate peels).

**Fig. 1:**
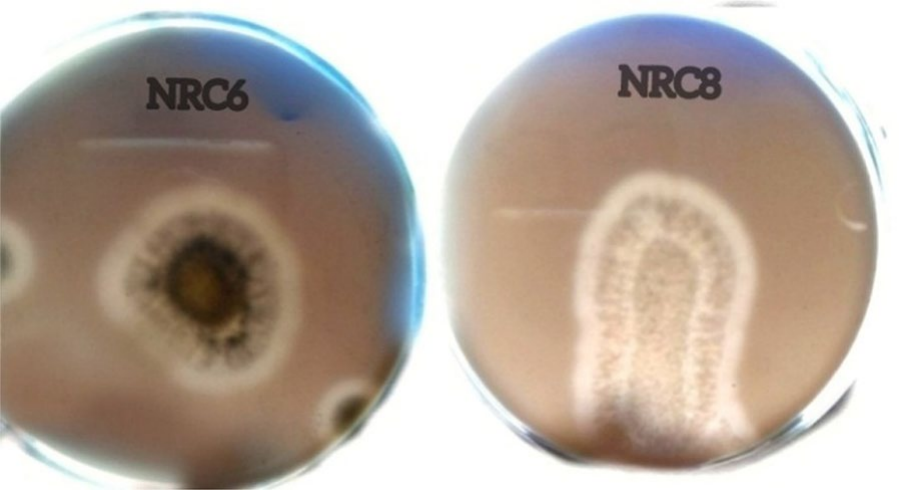
Tannolytic activity of different fungal isolates on agar plates

All fungal isolates were screened for their ability to produce tannase using a qualitative plate assay based on their ability to form a clear zone on agar plates containing tannic acid as a sole source of carbon. Among 15 fungal isolates screened for tannase activity, 13 fungal isolates grew and showed a clear zone around colonies in modified Czapek-Dox agar medium Czapek-Dox medium supplemented with tannic acid (Fig. 1).

### Quantitative assay test

Thirteen fungal isolates, had clear zone in plates were subjected to a quantitative assay test using modified Czapek-Dox medium containing tannic acid. It is clear that, all selected fungal isolates produced tannase in different proportions in a SSF. However, fungal isolate (NRC8) gave the highest tannase activity (46.71 U/ml) by SSF (Table 2).

**Table 2:** Quantitative screening of fungal isolates for the highest production of extracellular tannase

Fungi isolates	Tannase activity (U/ml)
NRC1	11.09
NRC2	21.28
NRC3	26.88
NRC4	33.6
NRC5	22.4
NRC6	39.2
NRC7	34.72
NRC8	46.71
NRC9	31.36
NRC10	24.64
NRC11	30.24
NRC12	33.60
NRC13	31.60

### Detection of aflatoxins in the culture filtrate of the selected fungal isolate

**Fig. 2:**
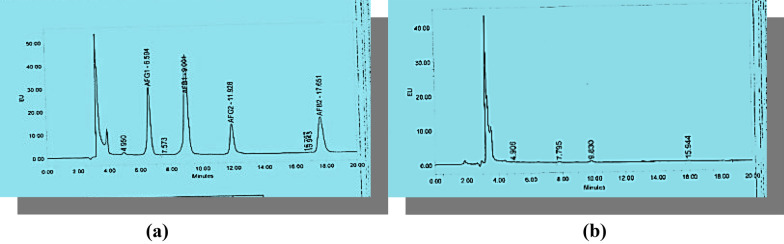
Detection of aflatoxins in the culture filtrate of isolate NRC8. **a** Stand sample, **b** isolate NRC8

Selected fungal isolate NRC8 was subjected to aflatoxins test. No aflatoxins (B_1_, B_2_, G_1_ and G_2_) were detected in the culture filtrate of the fungal isolate as shown in Fig. 2b in comparison with a standard aflatoxin as indicated in Fig. 2a.

### Molecular identification

**Fig. 3:**
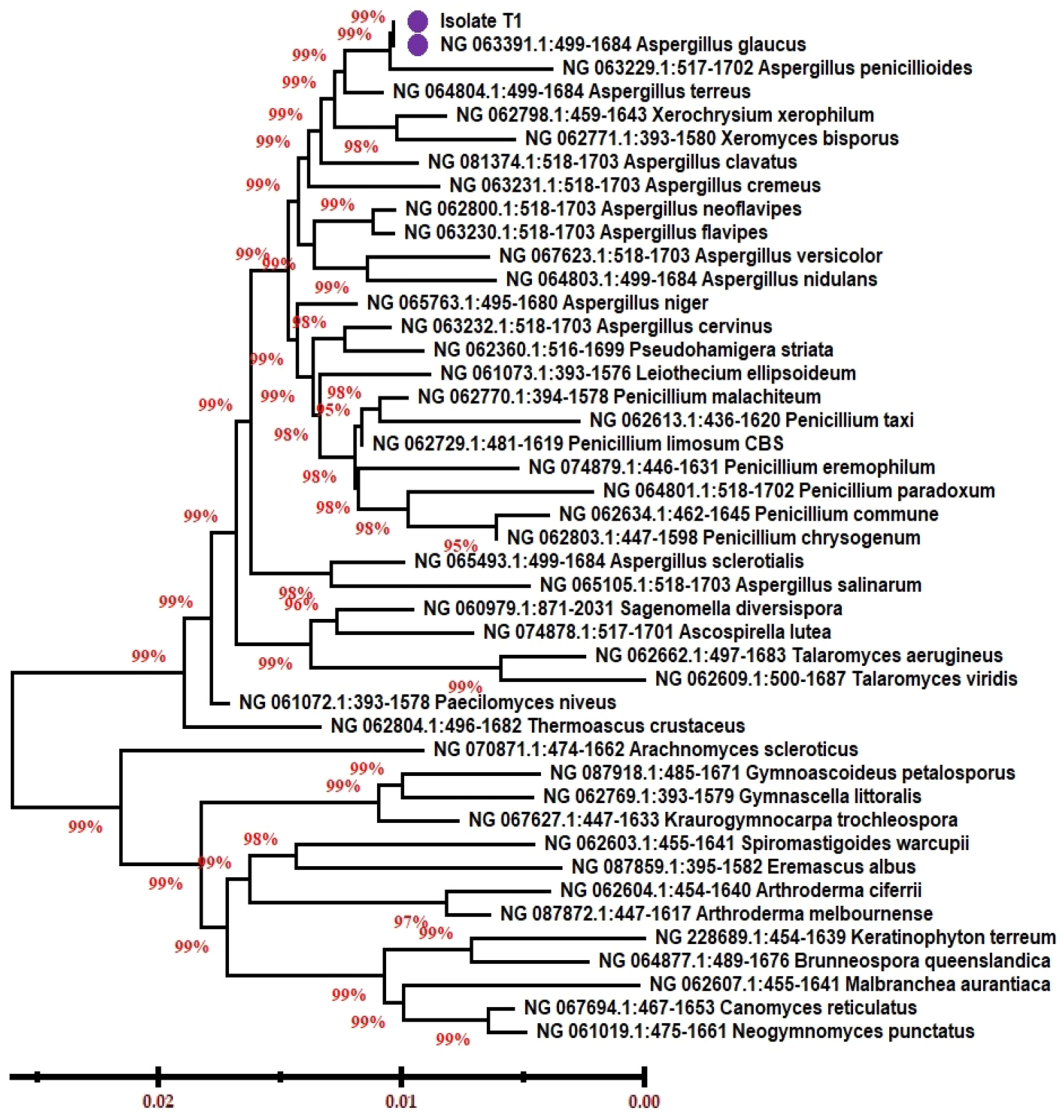
Phylogenetic tree of *Aspergillus glaucus* NRC8

The selected fungal isolate NRC8 was molecularly characterized and identified using 18S rRNA gene analysis using common primers. Using gene sequence library from NCBI database, data revealed approximately 98% similarity of 18S rRNA gene sequence of the isolate to the fungal strain *Aspergillus glaucus*. Various sequences taken from the GenBank database were used to build the phylogenetic tree to determine the phylogenetic position of the strain (Fig. 3) According to these morphological and molecular analysis, it was confirmed that the isolate NRC8 is closely allied to *Aspergillus glaucus* (GeneBank Accession Number_MZ19837).

### Optimization of tannase production by *Aspergillus glaucus*

#### Effect of different substrates on tannase production by *Aspergillus glaucus*

Different tannin-containing agricultural residuals (maize bran, rice bran, wheat bran, olive leaves, coffee husk, black tea waste, green tea waste, banana peels, tamarinds seed powder, pomegranate peels, potato peels) were tested as substrates for the production of tannase along with pure tannic acid as control. Data shown in Table 3 revealed that black tea powder was the optimal substrate to obtain highest tannase specific activity (2.97 U/mg), if compared with other substrates including pure tannic acid.

**Table 3:** Effect of different tannin-rich wastes on tannase production by *Aspergillus glaucus*

Substrates	Specific activity (U/mg)
Tannic acid (control)	2.23
Maize bran	0.93
Rice bran	1.05
Wheat bran	0.84
Olive leaves	0.88
Coffee	0.91
Black tea waste	2.97
Green tea	2.44
Banana peel	0.78
Tamarind	1.19
pomegranate peel	0.62
Potato peels	0.23

#### The optimum physiochemical parameters for tannase production by *Aspergillus glaucus*

Results shown in Table 4 showed the optimal physical and chemical factors for tannase production by *Aspergillus glaucus*. They are 75%, (5), 30 °C, 5 days, 7 g, 6 × 10^8^ spore/ml, and 0.2% of sodium nitrate for (moisture percentage, pH, temperature, incubation period, tea concentration, spore suspensions and the best nitrogen source), respectively.

**Table 4:** Effect of physiochemical parameters for tannase production by *Aspergillus glaucus*

Factors	Optimum value	Specific activity (U/mg)
Moisture percentage (%)	75	3.02
pH	5.0	3.18
Temperature (°C)	30	3.31
Incubation period (days)	5.0	3.48
Tea concentration (%)	7.0	3.55
Spore suspensions (spore/ml)	6 × 10^8^	3.70
Nitrogen source	NaNO_3_	3.81

### Response surface experimental approach using Box–Behnken design

After determination of the most four effective factors, RSM using Box–Behnken was conducted through 27 run orders for tannase production at 30 °C under SSF (Table 5). Specific tannase activity of each run was considered the response value (*Y*).

**Table 5:** Box–Behnken design

Run no.	*X1*: moisture ratio %	*X2*: initial pH	*X3*: substrate concentration	*X4*: sodium nitrate concentration	*Y*: tannase (U/mg)	
					Observed	Predicted
1	65	8	3	0.6	3.02	1.83125
2	65	8	6	1.0	2.94	3.27125
3	65	6	9	0.2	2.15	2.03417
4	65	4	9	0.6	2.15	2.56625
5	65	6	9	1.0	3.62	3.69750
6	100	6	6	1.0	2.32	1.96458
7	65	6	6	0.6	5.22	5.20333
8	30	6	6	0.2	0.42	0.00292
9	65	6	6	0.6	5.17	5.20333
10	65	4	3	0.6	2.37	2.14958
11	100	6	9	0.6	2.88	3.69125
12	30	4	6	0.6	1.32	1.77750
13	100	8	6	0.6	3.81	3.49750
14	65	4	6	1.0	3.33	2.79958
15	65	8	9	0.6	4.30	3.74792
16	65	6	3	0.2	1.79	1.85750
17	30	6	6	1.0	3.89	4.10625
18	100	6	3	0.6	1.48	2.74458
19	30	8	6	0.6	2.35	2.91417
20	30	6	3	0.6	1.42	1.23625
21	100	6	6	0.2	5.71	4.72125
22	65	8	6	0.2	1.40	2.55792
23	65	4	6	0.2	1.87	2.16625
24	100	4	6	0.6	4.19	3.77083
25	65	6	3	1.0	1.28	1.54083
26	30	6	9	0.6	3.26	2.62292
27	65	6	6	0.6	5.22	5.20333

As shown in Table 5, tannase productivity with practical and predicted results was clearly demonstrated. In addition, the analysis of variance (ANOVA) for the obtained results is also summarized in Table 6. According to ANOVA results, the significance of the model was achieved and confirmed by the *F* value, *P* value and lack-of-fit. In this regard, the *F* value and *P* value of the model was 4.30 and 0.002, respectively, which implies that the model is significant. In addition, the lack-of-fit was 1040.37 which indicates the model significance relative to the pure error. Furthermore, regression coefficient (*R*^2^) was used to show the fit of the model. It was observed that the model regression coefficient (*R*^2^) was close to 1.0 which means that all the points of the model can be predicted. The model predicted *R*^2^ was 0.893 for tannase which is in a considerable closing to the adjusted *R*^2^ of 0.86, respectively. In conclusion, the model adequate precision was 26 along with the previous results emphasized that the model can be established to navigate the design space.

**Table 6:** Analysis of variance

Source	DF	Adj SS	Adj MS	*F* value	*P* value
Model	14	43.4731	3.1052	4.30	0.002
Linear	4	10.9819	2.7455	3.80	0.032
Moisture%	1	4.9794	4.9794	6.89	0.022
pH	1	0.5590	0.5590	0.77	0.396
Substrate conc	1	4.0833	4.0833	5.65	0.035
NaNO_3_	1	1.3601	1.3601	1.88	0.195
Square	4	18.6367	4.6592	6.44	0.005
Moisture%*moisture%	1	6.5318	6.5318	9.03	0.011
pH*pH	1	6.5318	6.5318	9.03	0.011
Substrate conc.*substrate conc	1	12.3695	12.3695	17.11	0.001
NaNO_3_*NaNO_3_	1	10.4222	10.4222	14.42	0.003
2-way interaction	6	13.8545	2.3091	3.19	0.041
Moisture%*pH	1	0.4970	0.4970	0.69	0.423
Moisture%*substrate conc	1	0.0484	0.0484	0.07	0.800
Moisture%*NaNO_3_	1	11.7649	11.7649	16.27	0.002
pH*substrate conc	1	0.5625	0.5625	0.78	0.395
pH*NaNO_3_	1	0.0016	0.0016	0.00	0.963
Substrate conc.*NaNO_3_	1	0.9801	0.9801	1.36	0.267
Error		12	8.6756	0.7230	
Lack-of-fit	10	8.6739	0.8674	1040.87	0.001
Pure error		2	0.0017	0.0008	
Total		26	52.1487		

Regression equation of enzyme in uncoded units was as follows:

$${\text{Tannase}}\,{\text{specific}}\,{\text{activity}}\,\left( Y \right) = - 24.65 \, + \, 0.2458X1 + \, 3.37X2 + \, 1.671X3 + \, 16.66X4 - \, 0.000903X1*X1 - \, 0.2767X2*X2 - \, 0.1692X3*X3 - \, 8.74X4*X4 - \, 0.00504X1*X2 - \, 0.00105X1*X3 - \, 0.1225X1*X4 + \, 0.0625X2*X3 + \, 0.025X2*X4 + \, 0.413X3*X4.$$

According to the ANOVA results shown in Table 6, the *P* value of regression model is > 0.005. It indicates that the regression equation used to express the relationship between each variable and response value resulted in a very significant linear relationship between the dependent variable and each independent variable.

### Verification and validation experiment

**Fig. 4:**
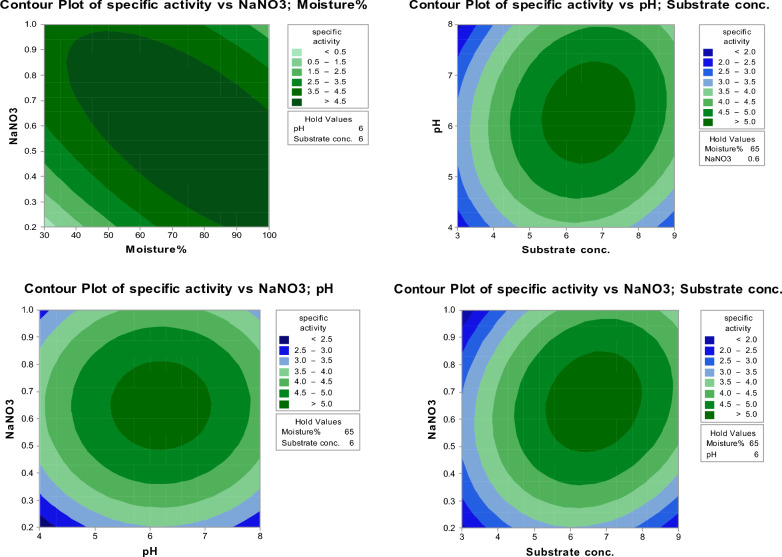
Contour line plots of the effect of cross-interaction among different variables on tannase production by *Aspergillus glaucus*

Generation of the Box–Behnken results was carried out by contour plot charts based on the regression equation to explore the response surface shape. These charts could clearly demonstrate the relationship between each variable factor in terms of the enzymatic productivity. As can be seen in Fig. 4, all plot charts having an open downward with different convex direction, implying potential tannase productivity. Moreover, the contour centers of the three response surfaces are sited within the set range indicating optimal conditions of model occur on the level of designed factors. The contour line also demonstrates oval shape and the interaction between NaNO3, pH and substrate conc., pH was significant.

After determination of the significant variables, verification of the investigated design would be created to confirm its validity. In this regard, validation test of tannase production has been running using Minitab-17. As can be seen in Fig. 4, the effective interaction between variables observed the best response for the tannase productivity, since the maximum yield of tannase was predicted by the analysis of combination between each variable with numerical optimization level.

**Fig. 5:**
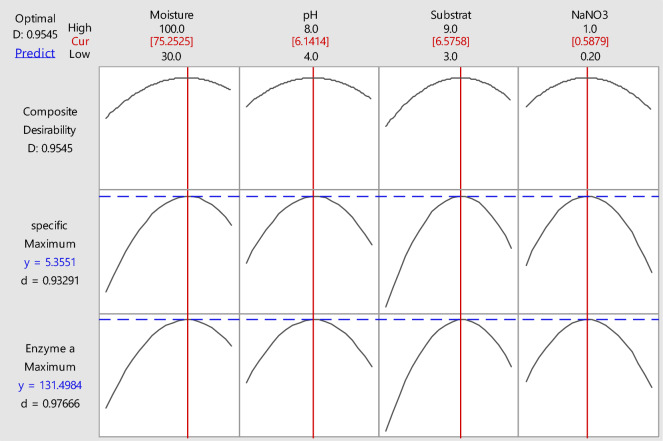
Response optimizer of tannase production

As a consequence of this analysis, optimized cultivation process could be determined precisely. Therefore, optimized cultivation conditions were as follows: moisture ratio (75.2525%), initial pH (6.1414), substrate concentration (6.5758 g/L) and NaNO3 (0.5879%); which was predicted to produce 5.3551 U/mg of tannase (Fig. 5). Interestingly, the practical tannase production (5.1992 U/mg) was found to be close to the predicted value. Accordingly, compatible between the practical and predicted results revealed the validity of the designated model.

### Production of gallic acid in *A. glaucus* cultured broth using black tea waste

The gallic acid content was estimated in the fermented culture broth. It was found that gallic acid concentration produced from SSF process of *A. glaucus* using black tea waste as substrate was 38.27 mg/ml.

### Bioconversion of tannin and gallic acid production by *A. glaucus* tannase

Applicability of the biosynthesized tannase from *A. glaucus* was studied in bioconversion of tannic acid to gallic acid. Thus, tannase enzyme was incubated with different concentration of tannic acid (50, 100 and 200 g/L) at two different temperatures (30 °C and 40 °C) under shaking conditions. Gallic acid concentration and both residual tannic acid concentration and enzyme activity were examined in the samples. Results (Table 7) illustrated that the bioconversion efficiency was higher at 40 °C than that at 30 °C. The tannic acid conversion efficiency was reduced when the tannic acid concentration became 200 g/L.

**Table 7:** Bioconversion of tannins and gallic acid production by *A. glaucus* tannase

Initial tannic acid concentration (g/L)	The total tannin content	Residual tannic acid concentration	Gallic acid content (mg/ml)	Tannan deposition (%)
*30 °C*				
50	33.6	25.97	37.03	22.71
100	89.6	71.09	28.34	20.66
200	179.2	149.55	23.17	16.55
*40 °C*				
50	33.6	21.90	51.33	34.82
100	89.6	59.27	39.92	33.85
200	179.2	140.39	24.08	21.66

## Discussion

*Aspergillus glaucus* is the most potent tannase producer in 15 isolated marine and terrestrial strains which showed a clear zone on the cultured medium referring to tannase production. This result agreed with Chhokar et al. (2010) who reported microorganisms have the ability to break down tannic acid and form a hydrolysis zone on the screening medium to indicate the presence of tannase. As reported (Spier and Gutterres 2019), the filamentous fungi have the ability to break down the ester and depside bonds found in tannic acid to generate gallic acid and glucose as hydrolysis products. Similarly, many studies indicated the ability of the isolated fungi on hydrosylation of tannic acid (de Melo et al. 2014; Kaur and Katyal 2018).

The SSF was superior cultivation method of *A. glaucus* for tannase production with highest specific activity (2.29 U/mg).This confirms the results recorded by Ahmed and Abou-Taleb (2019) that SSF of pomegranate peel led to an enhanced production of tannase activity and gallic acid yield by *A. niger *A8 which was 4.4-fold and 1.1-fold compared to the LSF and SmF, respectively. It goes to the advantages of using SSF over conventional SmF for enzyme production: 1. Simplicity of the medium using agro-industrial byproducts. 2. Low moisture level and low volume of medium per unit weight of substrate (Kumar et al. 2015).

Out of different tested natural sources of substrate (maize, rice bran, wheat bran, olive leaves, coffee husk, residual tea, green tea, banana peels, tamarinds seed powder, pomegranate peels, potato peels), residual tea was superior. This result makes a perfect match with the values reported in tea as substrate such as: 1. tannin concentration in tea (11–15%) (Khasnabis et al. 2015); 2. diverse nutritional constituents (sugars, amino acids, caffeine, polyphenol, lipids, organic acids and minerals) which stimulate both microbial growth and tannase production and 3. high water retention capacity of the tea makes the growth medium is highly available to the fungal mycelia (Sharma et al. 2008; Beniwal et al. 2013).

Low moisture content (75%) was favored by *A. glaucus* for tannase production. It can be referred to the low water activity needs of fungi. Filamentous fungi are known to grow at water deficient substrates with low moisture content such as bark of trees and dry leaves. As it was concluded, poor oxygen availability is resulted from high moisture contents which affects the fungal growth and tannase productivity (Hölker et al. 2004; Kumar et al. 2007). Similarly, low pH value of 5.0 enhanced the tannase production. The pH values around pH 5.0 have been reported to be optimal for tannase production from *Aspergillus niger* (ATCC 16620 and Aa-20) and *Aspergillus ruber* (Sabu et al. 2005; Treviño-Cueto et al. 2007).

Interestingly, pH value of 5.0 was also optimal for the activity of the partial purified tannase. In agreement with this finding, the optimal activity of the purified tannase from both *Penicillium notatum* NCIM 923 and *K. pneumoniae* MTCC 7162 was obtained at pH 5.0 (Gayen and Ghosh 2013; Sivashanmugam and Jayaraman 2013). It was previously reported that tannase is an acidic enzyme, however, the influence of the pH on the enzymatic activity depends mainly on: 1. structural changes of the protein after amino acids ionization and 2. type of the amino acids (both protonated and deprotonated) at the enzyme active site (Beniwal et al. 2013; Wakil et al. 2020).

Incubation of fungi at 30 °C was optimal for tannase production, higher temperature led to reduction in the activity of the produced tannase. This latter observation may be due to high sporulation rate which is induced at high temperatures and hinder the growth of fungal mycelia (Sharma et al. 2014). Correspondingly, the optimal production of tannase enzyme from *Aspergillus fumigates* MA and *Aspergillus oryzae* was also obtained at temperature span of 30–35 °C according to the type of substrate used in the SSF (Beniwal et al. 2013).

A decline in the fungal growth and tannase productivity was observed above 6 days which coincidence with entry of the decline phase of the fungal growth. In connection, Akhavan Sepahy et al. (2011) related this decline to micro- and macro-nutrients consumption from the production medium which results in pH change which consequently affects the fungal growth and productivity. Alike to this result, both *A. niger* A8 and *T. viride* had the highest capabilities of degradation of tannin-rich materials, pomegranate peel and banana peel, in SSF at 30 °C for 6 days (Ahmed and Abou-Taleb 2019).

Tea powder was a potent substrate for tannase production. Yet, enzyme activity was decreased by adding above seven grams of tea powder in the growth medium. It was found that high concentration of tannic acid is known to be a repressor in most of the former studies. However, it was reported that *A. niger* was able to grow in a high concentration of tannic acid (up to 100 g/L) in SSF without any adverse effect on growth or tannase production (Rodríguez-Durán et al. 2011; Beniwal et al. 2013). In connection, among different nitrogen sources, NaNO_3_ supported maximal fungal growth and tannase production. Confirmatory to this result, Wakil et al. reported NaNO_3_ as the best nitrogen source for maximum tannase production (15.88 U/ml) by different fungal isolates (Wakil et al. 2020).

Although gallic acid exists naturally in plants, they are produced by microbial hydrolysis of tannin using the enzyme tannase because of their wide application and demands industrially. In connection, Abd-Elmotey et al. (2022) reported production of gallic acid ranging from 33.1 to 255.4 mg/ml while testing *Aspergillus niger* SWP33 and *Penicillium griseoroseum *T11 for tannase production using agricultural wastes under SmF and SSF. Similarly, Olaleye and Omotayo (2022) who found that* A. versicolor* was able to produce a maximal gallic acid concentration of 9.42 mg/ml using acacia as substrate.

Higher and constant efficiency of tannin bioconversion is achieved at 40 °C in given incubation time. This was also postulated by the result showed the thermal stability of *A. glaucus* tannase at 40 °C. The tannic acid conversion efficiency was reduced by raising the tannic acid concentration to 200 g/L. There are no recorded reports on the stability of tannase at high tannic acid concentrations (above 50 g/L). This may be due to limitations of the substrate diffusion and mass transfer at high concentration (Abd-Elmotey et al. 2022).

## Conclusion

This study introduces a local fungal strain, *Aspergillus glaucus*, able to bio-produce tannase enzyme by SSF process using the residual tea low-cost substrate. Production of gallic acid occurred, as a byproduct from hydrolysis of tannin with *Aspergillus glaucus*, at cultured medium. Tannase enzyme is industrially high-added-value particularly in food industry. Maximum tannase production was obtained after 144 h of the incubation at 30 °C and pH 5.0. At our research, using SSF process for tannase production by *A. glaucus* activity reached 100.8 U/ml, which was 1.23 times higher than enzyme obtained in SmF process (81.76 U/ml). Also, our results are higher than which obtained under SSF using agro-industrial wastes for tannase production by *P. montanense* where tannase activity reached 41.64 U/mL at optimal condition (Juliana et al. 2014). The maximum specific activity of *A. glaucus* tannase under SSF was (5.37 U/mg) that result is more than results of Ahmed and Abou-Taleb (2019) who indicated that *A. niger* A8 and *T. viride* gave a high specific activity of tannase (from 8.08 to 10.95 U/mg) on pomegranate and banana peels supplemented with corn steep liquor under SSF system. Higher and constant efficiency of tannin bioconversion is achieved in given incubation time by using *A. glaucus* tannase at 40 °C.

## Data Availability

Not applicable.
